# Case report: Neuroacanthocytosis associated with novel variants in the *VPS13A* gene with concomitant nucleotide expansion for CANVAS and assessment with osmotic gradient ektacytometry

**DOI:** 10.3389/fnins.2024.1409366

**Published:** 2024-10-02

**Authors:** Martin Paucar, Josephine Wincent, Charlotta Rubin, Kevin Peikert, Josefin Kyhle, Stellan Hertegård, Riita Möller, Soheir Beshara, Per Svenningsson

**Affiliations:** ^1^Department of Clinical Neuroscience, Karolinska Institutet, Stockholm, Sweden; ^2^Department of Neurology, Karolinska University Hospital, Stockholm, Sweden; ^3^Department of Molecular Medicine and Surgery, Karolinska Institutet, Stockholm, Sweden; ^4^Department of Clinical Genetics, Karolinska University Hospital, Stockholm, Sweden; ^5^Department of Clinical Nutrition, Karolinska University Hospital, Stockholm, Sweden; ^6^Translational Neurodegeneration Section “Albrecht Kossel,” Department of Neurology, University Medical Center Rostock, University of Rostock, Rostock, Germany; ^7^Center for Transdisciplinary Neurosciences Rostock, University Medical Center Rostock, Rostock, Germany; ^8^United Neuroscience Campus Lund-Rostock, Rostock, Germany; ^9^Department of Speech and Language, Karolinska University Hospital, Stockholm, Sweden; ^10^Department of Clinical Science, Intervention and Technology, Karolinska Institutet, Stockholm, Sweden; ^11^Department of Otolaryngology, Karolinska University Hospital, Stockholm, Sweden; ^12^Department of Medical Biology and Biostatistics, Karolinska Institutet, Stockholm, Sweden; ^13^Department of Clinical Chemistry, Karolinska University Hospital, Stockholm, Sweden

**Keywords:** neuroacanthocytosis, ektacytometry, *VPS13A*, *VPS13A* disease, feeding dystonia

## Abstract

**Background and objectives:**

The diseases historically known as neuroacanthocytosis (NA) conditions include *VPS13A* disease (formerly chorea-acanthocytosis) and *XK* disease (formerly McLeod syndrome). Here we report a patient with a hyperkinetic syndrome associated with variants in *VPS13A* with a concomitant homozygous nucleotide expansion in Replication factor C, subunit 1 (*RFC1*) and evaluate the role of ektacytometry for the assessment of acanthocytes.

**Methods:**

Investigations included clinical assessments, neuroimaging studies, laboratory analyses, blood smears, ektacytometry, psychometric evaluation, and genetic analyses. Using ektacytometry, an osmoscan curve is obtained yielding a diffraction pattern as a measure of average erythrocyte deformability from circular at rest to elliptical at a high shear stress. The pattern allows the derivation of several parameters (mainly EI-max, O-min and O-Hyper points). Samples from two other patients with genetically proven *VPS13A* disorder and *XK* disease and varying numbers of acanthocytes as well as from a fourth with acanthocytosis due to liver failure were also analyzed.

**Case presentation:**

The patient has impulsivity, chorea and disabling feeding dystonia refractory to treatment and 15% acanthocytes in peripheral blood. Genetic workup revealed compound heterozygous variants c.1732_1733del; p.(V578Ffs*9) and c.8282C > A, p.(S2761*) in *VPS13A* with absence of chorein in the blood, the latter variant is novel. In addition, he harbors a homozygous nucleotide expansion in the *RFC1* gene, reported in cerebellar ataxia, neuropathy, vestibular areflexia syndrome (CANVAS). However, the patient does not display ataxia yet. Ektacytometry revealed significantly reduced erythrocyte deformability in this patient and in another man with *VPS13A* disease. In contrast, the patient with *XK* disease had 2% acanthocytes and mild abnormalities on ektacytometry. In the three cases, ektacytometry yielded a specific pattern, different from acanthocytosis due to liver failure.

**Conclusion:**

Pathogenicity of the *VPS13A* variants is confirmed by absence of chorein, long-term follow up is required to evaluate any synergistic impact of for the underlying CANVAS mutation. New generation ektacytometry provides an objective measurement of erythrocytes’ rheological properties and may serve as a complement to blood smears. Finally, ektacytometry’s ability to detect deformability of erythrocytes in NA seems to depend on the degree of acanthocytosis.

## Background

*VPS13A* disease (formerly known as chorea-acanthocytosis) and *XK* disease (formerly McLeod syndrome) are the conditions historically known as the core neuroacanthocytosis (NA) syndromes (Walker et al., 2023; Walker and Danek, 2021; Peikert et al., 2002; Jung et al., 2004). The percentage of acanthocytes in both *VPS13A* disease and *XK* disease is variable (5–50%) under the course of disease, > 6.3% acanthocytes in peripheral blood is considered abnormal. To reduce the risk for false positive and negative cases, acanthocytosis is assessed with a specific protocol (Storch et al., 2005). The red blood cells membrane properties are determined by membrane structure, which in turn, identifies membrane deformability, mechanical stability and permeability. These rheological properties can be measured by osmotic gradient ektacytometry. Data on ektacytometry in NA syndromes is still very limited (De Franceschi et al., 2014; Cluitmans et al., 2015; Lazari et al., 2020; Ballas et al., 1990; Hernández et al., 2024).

## Patient and methods

Investigations included clinical assessments, neuroimaging studies, laboratory analyses, blood smears, ektacytometry, psychometric evaluation, and genetic analyses. A Western blot analysis for chorein, with two different antibodies (Anti-VPS13A, HPA021662, Sigma-Aldrich, rabbit and Anti-VPS13A, PA5-54483, Invitrogen, rabbit), was performed according to the protocol described by Dobson-Stone et al. (2004). Briefly, one antibody targets an epitope located in middle of the protein (Invitrogen), and the second one targets an epitope in the C-terminus (Sigma). Absence of bands with both antibodies reflects lack of chorein expression, even of a truncated protein. Blood samples from a man with genetically proven *VPS13A* disease previously reported by Paucar et al. (2015), from a man with *XK* disease harboring the recurrent variant c.397C > T; p.R133X in *XK* (Dotti et al., 2000; Klempír et al., 2008; Murakami et al., 2019) and from a woman with liver failure and acanthocytosis were also included for comparison.

## Blood smear and ektacytometry

In all cases, the presence of acanthocytes was assessed according to the protocol for wet blood smears proposed by Storch et al. (2005). Erythrocytes were also studied using new generation osmotic gradient ektacytometry (LoRRca^®^ Maxis; RR Mechatronics). By mixing low and high osmolar solutions, an osmotic gradient was established, and the laser diffraction pattern was recorded. Few microliters of blood were suspended in 5 ml of isotonic Polyvinylpyrrolidone solution (PVP) (RR Mechatronics) and mixed carefully. Osmoscan curve was performed, and the following variables were obtained: the minimal osmolality (O-min), where 50% of RBC are lysed in a hypoosmotic environment and its corresponding minimal elongation index (EI-min), the maximal elongation index (EI-max) at optimal osmolality (O-max), the hyperosmotic osmolality (O-hyper), where half of the maximal elongation index (EI-hyper) is reached, and the area under the osmoscan curve (AUC) was calculated.

## Massive parallel sequencing and Sanger sequencing

Massive parallel sequencing (MPS) of the patient was performed using a 30× PCR-free paired-end WGS protocol on an Illumina NovaSeq 6000 platform as described previously (Magnusson et al., 2020). A gene panel of 956 genes associated with movement disorders and neuromuscular disease was analyzed. The variants were prioritized based on conservation, frequency in internal and public databases, and pattern of inheritance. The ranked variants were then visualized in the Scout analysis platform (Stranneheim et al., 2021). Both *VPS13A* variants, confirmed in the patient by PCR and Sanger sequencing, were segregated in the parents. The Sanger sequencing was performed by standard methods on an ABI 3730 PRISM^®^ DNA Analyzer. Primer sequences available upon request. The homozygous *RFC1* expansion was confirmed by PCR amplification covering the repeat region.

## Results

### Case presentation

**FIGURE 1:**
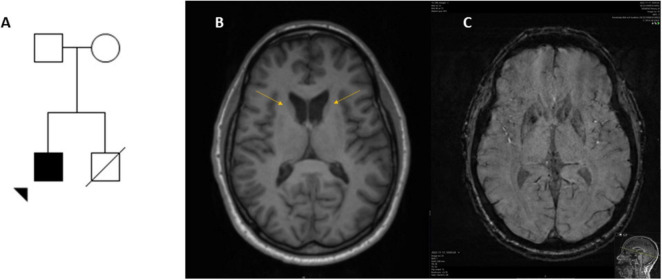
Pedigree and neuroimaging of a man with *VPS13A* disease and carrier for a homozygous nucleotide expansion in *RFC1*. The patient (arrowhead) is of Finnish descendancy, each parent carries a variant in *VPS13A* **(A)**. Neuroimaging at age 35 years, T2 weighted axial image **(B)** displaying caudate atrophy (arrows) and subsequent widening of the ventricles and a SWI sequence with increased iron levels in the same structure **(C)**. Motion artifacts limited assessment of the putamen.

This is a 36-year-old man (index case, patient A) born to healthy and non-consanguineous Finnish parents, the pedigree is presented in Figure 1A. One sibling, affected by obesity, hypertension, and reduced systolic function had limited compliance to medication, died suddenly at age 31 years some years before the index case came to our center for evaluation. The cause of death could not be determined, his DNA was not available for analyses. The patient was referred due to personality change, impulsivity, slurred speech, progressive involuntary movements, and severe eating difficulties. The patient had involuntary lingual movements with tongue protrusion induced by eating, with frequent biting of the lips and tongue. The patient has been using dental guards but bit off these devices. Fiberoptic endoscopic evaluation of swallowing revealed oropharyngeal dysphagia. The patient reported weight loss, involuntary leaning of the trunk backward but denies falls, he has not required walking aids, and has been able to engage in winter sports (skiing and skating on ice). Upon exam he displayed moderate chorea in the face, perioral region and extremities as well as feeding and truncal dystonia. There were no signs of lingual motor impersistence. He could walk and run and performed tandem gait without difficulties. There were no signs of dysmetria, or nystagmus, vestibular-ocular reflex (VOR) was normal on video head impulse test (vHIT). He had areflexia, plantar responses were flexor; muscle tone, strength and sensation were normal. There was no evidence of muscle atrophy either. Eye movements were characterized by broken smooth pursuit and mild slowness of vertical saccades. Horizontal optokinetic nystagmus (OKN), amplitude and speed on vertical OKN were normal. The last exam yielded 29 points in the Unified Huntington’s Disease Rating Scale-Total Motor Score (UHDRS-TMS) protocol. Several drugs (different neuroleptics alone and combined, tetrabenazine, trihexyphenidyl, and dantrolene) were tried for feeding dystonia without any benefit. Botulinum injections were applied to the pterygoid muscles but not to the tongue base since this pose the risk of worsening his dysphagia. The patient was offered a gastrostomy, but he decided to delay the procedure. His last BMI was 23.5, his diet consists almost exclusively of oral nutrition supplements. The patient declined to use psychotropic medicines. The cognitive evaluation revealed difficulties performing the Luria test, a recent neuropsychological assessment found clear variations ranging from significantly below average-to-average performance. Clearly low performances were found in verbal episodic memory, both learning and retrieval of verbal material as well as verbal working memory. Likewise, executive abilities, such as word flow and flexibility (ability to switch between concepts in visual tempo-demanding tasks) were below average. The work pace in various tasks was also slower than expected. There were no signs of hemolysis except a mild reduction of haptoglobin levels. CK levels and EMG were normal, a neurography demonstrated incipient sensory neuropathy whereas his EEG was normal (Clinical findings are summarized in Table 1). The MRI of the brain shows caudate atrophy and increased iron accumulation in this structure (Figures 1B, C), his cardiac evaluation (echocardiography and ECG) was unremarkable.

**TABLE 1:** Summary of clinical findings in a man with pathogenic biallelic new variants in *VPS13A* and a homozygous nucleotide expansion in *RFC1*.

Parameter	Findings
Motor features	Chorea and severe feeding dystonia
Other neurological features	No
Psychiatric features	Impulsivity
Cognition	Mild executive difficulties
Seizures	No
Genetic variants	*VPS13A* c.1732_1733, p.(Val578Phefs*9)^a^ c.8282 C > A, p.(Ser2761*)^b^ Homozygous pentanucleotide expansion in *RFC1*
Diagnosis	*VPS13A* disease
Ektacytometry	Clearly abnormal
Degree of acanthocytosis (%)	15%
ENeG/EMG	Abnormal values on ENeG
Neuroimaging	Caudate atrophy Increased iron levels in the caudate nuclei
Hemolysis	No^c^
CK	Normal
Myoglobulin	Normal
Transaminases	Normal

AST, aspartate aminotransferase; BG, basal ganglia; ENeG, electroneurography; ND, no determined.

^a^This variant is found in 2 individuals (Finns) in gnomAD, CADD = 32.

^b^This variant is absent in gnomAD. CADD = 45.

^c^Only haptoglobin was mildly reduced, but other tests for hemolysis (blood cell count, reticulocytes, lactate dehydrogenase, and bilirubin) were all normal.

### Genetics and biochemistry

The patient was initially investigated for Huntington’s disease (HD) but lacked pathological CAG expansions in the *HTT* gene. MPS identified compound heterozygous truncating variants in *VPS13A*. The variant c.1732_1733del; p.(V578Ffs*9) (CADD 32) was inherited from the patient’s father and was previously reported in the heterozygous state in two individuals (Finns) in gnomAD v2.1.1 corresponding to a carrier frequency of 1/11000. The second variant c.8282C > A, p.(S2761*), (CADD 45) was inherited from the patient’s mother and was absent from gnomAD v2.1.1 Chorein/VPS13A protein was not detected by Western blot (Supplementary Figure 1). In addition, the patient carries a homozygous nucleotide expansion in the Replication Factor C, Subunit 1 (*RFC1*) gene.

### Blood smears, ektacytometry and blood count

**FIGURE 2:**
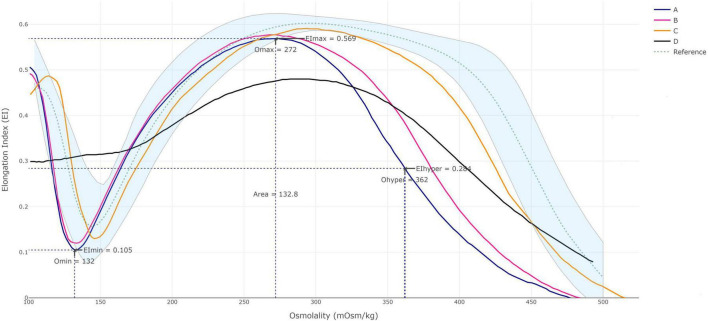
Ektacytometry data. Osmoscan profiles in the patient reported here with *VPS13A* disease **(A)**; another male patient with *VPS13A* disease with 12% acanthocytes **(B)**; a man with *XK* disease with 2% acanthocytes **(C)**; and in a female patient with liver failure and about 50% acanthocytes **(D)**. Continuous line represents the deformability of each patient and shaded area the control group. The patient samples were analyzed as single measurement each, but data are shown to be comparable with those previously presented (Ballas et al., 1990). The control group was established using data from 22 healthy controls and further verified using samples from additional 31 healthy subjects. Samples from 10 patients diagnosed with hereditary spherocytosis were included in the evaluation to discriminate the two different groups. Precision and sample stability testing were run according to internal laboratory procedures, the method was verified during spring 2023.

In blood smears from patient A, 15% acanthocytes were found, and ektacytometry revealed significantly reduced deformability in erythrocytes (Figure 2, curve A). Similar results were shown in another male patient with *VPS13A* disease (Hernández et al., 2024) with a similar count of acanthocytes in peripheral blood (Figure 2, curve B). In contrast, ektacytometry for the patient with *XK* disease, harboring 2% acanthocytes, revealed only mild abnormalities (Figure 2, curve C). In both cases, the most affected measurement in ektacytometry was the “O hyper” point. In contrast, acanthocytosis due to alcoholic liver failure yielded a completely different curve (Figure 2, curve D). Data from complete blood count are presented in Table 2. The genetic data was further studied regarding variants in the *PIEZO1* and *SPTA1* genes, that may yield altered curves. No pathogenic variants in either gene were found in patient A or for patient D (with advanced liver cirrhosis). Patients B and C went through targeted genetic testing for *VPS13A* and *XK* disease only, thus testing for other genetic erythrocyte membrane defects than for NA syndromes was not part of their work-up.

**TABLE 2:** Complete blood count of the reported cases.

Parameter and diagnosis	*VPS13A* disease (patient A)	*VPS13A* disease (patient B)	*XK* disease (patient C)	Liver cirrhosis (patient D)	Reference range
B-Hb	146	135	155	54	134–170 g/L
B-RBCs	4.9	4.5	4.6	1.6	4.2–5.7 10^12^/L
B-Hct	0.45	0.37	0.42	0.16	0.39–0.50
B-MCV	92	85	89	103	82–98 fL
Erc(B)-MCH	30	31	33	34	27–33 pg
Erc(B)-MCHC	324	365	369	338	317–357 g/L
Erc(B)-RDW	< 18	< 18	< 18	> 18*	< 18%
Reticulocyte count	142*	60	104	236*	28–115 10^9^/L
Leukocyte count	5.7	4.5	8.6	25.5*	3.5–8.8 10^9^/L
Platelet count	159	178	307	129*	145–348 10^9^/L

Erc(B)-RDW > 18% is considered pathologic and is reported as anisocytosis. Patient A is the case described in this work.

## Discussion

This is the first time a patient of Finnish origin is reported as affected by a *VPS13A* disease. Both *VPS13A* variants reported here are new in the context of *VPS13A* disease. Homozygous nucleotide expansions in *RFC1* are the underlying cause of cerebellar ataxia, neuropathy and vestibular areflexia syndrome (CANVAS). In this case, the only feature suggesting a *RFC1*-related disorder was incipient neuropathy. On the other hand, neuropathy is a common trait among patients with *VPS13A* disease. Long-term follow up is warranted to determine any synergistic effect of this dual pathology. Feeding dystonia, a hallmark of *VPS13A* disease, is particularly challenging to treat (Bader et al., 2010) as reported here. The risk for cardiomyopathy and ventricular arrhythmias in *XK* disease is well known, recently a study demonstrated that cardiomyopathy can also be a feature in *VPS13A* disease (Quick et al., 2021). The index case’s late brother who died suddenly and unexpectedly did not have any known neurological symptoms according to his relatives, a genetic investigation was not possible to carry out. Normal CK level in our patient is of note since this protein is elevated in the majority of patients with *VPS13A* disease (Peikert et al., 2002).

The morphology of erythrocytes is maintained by membrane lipids, proteins, and spectrin–actin membrane-skeleton. Cellular membranes comprise lipid bilayers that consist of glycerophospholipids, sphingolipids and cholesterol where phosphatidylcholine, phosphatidylethanolamine and phosphatidylserine (PtdSer) are among the major glycerophospholipids as well as phosphatidylserine (PtdIns) as one of the relatively minor glycerophospholipids (Sakuragi and Nagata, 2023).

The VPS13 protein family members (A–D) have been recently identified to be located at membrane contact sites acting in bulk lipid transfer (Leonzino et al., 2021). Mutations in the corresponding genes for the *VPS13* protein family members are of clinical importance as mutations in each protein lead to a specific neurological disorder (Peikert et al., 2002). However, acanthocytosis has only been identified in *VPS13A* disease giving rise to remarkable phenotypic similarities with *XK* disease, that is a genetically distinct disorder (Walker et al., 2023). XK on the other hand, was described as a transmembrane protein linked to the Kell protein by a disulfide bond in erythrocytes. XK and Kell seem, though, not linked in both brain and skeletal muscle (Spesivtseva et al., 2023). XKr8, however, was shown to function as a lipid scramblase and its role in apoptotic PtdSer exposure was subsequently identified (Suzuki et al., 2014). The reduced levels of PtdSer subspecies in the inner membrane leaflet of McLeod erythrocytes (with absent XK protein) had been already described by Redman et al. (1989). Further, VPS13A participates in regulating the phosphorylation of PtdIns on the plasma membrane of the erythrocytes. The level of PtdIns regulates the interaction between the plasma membrane and membrane-skeleton (Park et al., 2015).

Studying the pathophysiology of diseases due to mutations of the VPS13 protein family members, other lipid transfer proteins, and proteins related by a common function, such as XK, may indicate that these are all part of a group of disorders with a common mechanism of impaired bulk lipid transport (Walker et al., 2023).

The mechanism/s of the abnormal red cell membrane structure that results in acanthocytosis in *VPS13A* disease and *XK* disease is/are not fully understood. Several hypotheses are postulated among which is the disturbances in the phosphorylation-controlled binding between the integral membrane protein complexes and the membrane-skeleton (De Franceschi et al., 2014) and of depolymerized cortical actin due to absence or significant reduction in chorein encoded by *VPS13A* (Föller et al., 2012).

However, it now appears likely that VPS13 and XK proteins are functionally linked. At the plasma membrane, VPS13A interacts with the scramblase XK. This complex may be involved in lipid transport between the endoplasmic reticulum and plasma membrane as well as lipid scrambling between the two leaflets of the plasma membrane (Guillén-Samander et al., 2022). Erythrocytes lack organelles when circulating in peripheral blood, however, it is possible that abnormal lipid metabolism and organellar transfer during maturation (erythropoiesis) affects their membrane composition.

This complex function in membrane lipid homeostasis and survival resulting perhaps from deficient PtdSer and/or PtdIns exposure at the plasma membrane may explain the phenotypic similarities between the two disorders including formation of acanthocytes (Walker et al., 2023).

Ektacytometry is a widely used tool for assessing erythrocyte deformability and hydration status, giving a different pattern for each type of erythrocyte membrane defects (Da Costa et al., 2016; Andolfo et al., 2016). The significant change in ektacytometry data was mostly observed in the left shift of the O-hyper point, which reflects the stiffness of erythrocytes. A mild decrease in deformability was also noted in index patient as well as patient with neuroacanthocytosis with similar acanthocyte percentage in peripheral blood. The changes seen were closely related to the number of acanthocytes identified in patients with *VPS13A* disease and *XK* disease. These changes were, however, not reflected on hematological parameters. Rheologic abnormalities on four patients with *XK* disease reported by Ballas et al. (1990) are similar to our findings. Reichel et al. (2022) also found reduced erythrocyte deformability in *VPS13A* disease by means of microfluidic techniques. Data on ektacytometry for *VPS13A* disease and pantothenate kinase-associated neurodegeneration (PKAN), another condition also associated with acanthocytosis, is also limited (De Franceschi et al., 2014; Cluitmans et al., 2015). A report on ektacytometry of neuroacanthocytosis cases did not provide details on underlying genotype (Lazari et al., 2020). In contrast, spur cell anemia in liver failure is thought to be due to exogenous factors not related to membrane structure since transfused cells tend to undergo similar morphological alterations as well (Shah et al., 2023). That explains the fact that changes observed in ektacytometry displayed a completely different pattern in acanthocytosis caused by liver failure.

Ektacytometry is an objective tool to evaluate erythrocyte deformability. Our report, despite cross-sectional single measurements of few patients, adds on the utility of this tool for assessment of patients with *VPS13A* and *XK* diseases (Walker and Danek, 2021). This tool may constitute a promising complement especially when blood smears are inconclusive. Taken together, our data suggest an ektacytometry-based fingerprint for *VPS13A* disease, but larger studies are required to validate our findings.

## Patient perspective

The patient with *VPS13A* disease reported here provided oral and written consent for this work in the context of research approved by the Swedish Ethical Review Authority. The course of disease is progressive and feeding dystonia is highly disabling. Gastrostomy in a near future, is a reasonable and necessary option for this patient. The other patients whom blood was tested for acanthocytosis did also provide oral and written consent.

## Data Availability

The original contributions presented in this study are included in this article/Supplementary material, further inquiries can be directed to the corresponding author.

## References

[B1] Andolfo I. Russo R. Gambale A. Iolascon A. (2016). New insights on hereditary erythrocyte membrane defects. *Haematologica* 101 1284–1294. 10.3324/haematol.2016.142463 27756835 PMC5394881

[B2] Bader B. Walker R. Vogel M. Prosiegel M. McIntosh J. Danek A. (2010). Tongue protrusion and feeding dystonia: A hallmark of chorea-acanthocytosis. *Mov. Disord.* 25 127–129. 10.1002/mds.22863 19938148

[B3] Ballas S. Bator S. Aubuchon J. Marsh W. Sharp D. Toy E. (1990). Abnormal membrane physical properties of red cells in McLeod syndrome. *Transfusion* 30 722–727. 10.1046/j.1537-2995.1990.30891020333.x 2219261

[B4] Cluitmans J. Tomelleri C. Yapici Z. Dinkla S. Bovee-Geurts P. Chokkalingam V. (2015). Abnormal red cell structure and function in neuroacanthocytosis. *PLoS One* 10:e0125580. 10.1371/journal.pone.0125580 25933379 PMC4416783

[B5] Da Costa L. Suner L. Galimand J. Bonnel A. Pascreau T. Couque N. (2016). Society of hematology and pediatric immunology (SHIP) group; French society of hematology (SFH). Diagnostic tool for red blood cell membrane disorders: Assessment of a new generation ektacytometer. *Blood Cells Mol. Dis.* 56 9–22. 10.1016/j.bcmd.2015.09.001 26603718 PMC4811191

[B6] De Franceschi L. Bosman G. Mohandas N. (2014). Abnormal red cell features associated with hereditary neurodegenerative disorders: The neuroacanthocytosis syndromes. *Curr. Opin. Hematol.* 21 201–209. 10.1097/MOH.0000000000000035 24626044

[B7] Dobson-Stone C. Velayos-Baeza A. Filippone L. Westbury S. Storch A. Erdmann T. (2004). Chorein detection for the diagnosis of chorea-acanthocytosis. *Ann. Neurol.* 56 299–302. 10.1002/ana.20200 15293285

[B8] Dotti M. Battisti C. Malandrini A. Federico A. Rubio J. Circiarello G. (2000). McLeod syndrome and neuroacanthocytosis with a novel mutation in the XK gene. *Mov. Disord.* 15 1282–1284. 11104227 10.1002/1531-8257(200011)15:6<1282::aid-mds1042>3.0.co;2-2

[B9] Föller M. Hermann A. Gu S. Alesutan I. Qadri S. Borst O. (2012). Choreinsensitive polymerization of cortical actin and suicidal cell death in chorea-acanthocytosis. *FASEB J.* 26 1526–1534. 22227296 10.1096/fj.11-198317

[B10] Guillén-Samander A. Wu Y. Pineda S. García F. Eisen J. Leonzino M. (2022). partnership between the lipid scramblase XK and the lipid transfer protein VPS13A at the plasma membrane. *Proc. Natl. Acad. Sci. U.S.A.* 119:e2205425119. 10.1073/pnas.2205425119 35994651 PMC9436381

[B11] Hernández C. Peikert K. Qiao M. Darras A. de Wilde J. Bos J. (2024). Osmotic gradient ektacytometry - a novel diagnostic approach for neuroacanthocytosis syndromes. *Front. Neurosci.* 18:1406969. 10.3389/fnins.2024.1406969 39091345 PMC11292800

[B12] Jung H. Danek A. Walker R. Frey B. Peikert K. (2004). “McLeod neuroacanthocytosis syndrome,” in *GeneReviews^®^ [Internet]*, eds Adam M. Feldman J. Mirzaa G. Pagon R. Wallace S. Bean L. (Seattle, WA: University of Washington). 20301528

[B13] Klempír J. Roth J. Zárubová K. Písacka M. Spacková N. Tilley L. (2008). The McLeod syndrome without acanthocytes. *Parkinsonism Relat. Disord.* 14 364–366. 17870653 10.1016/j.parkreldis.2007.07.011

[B14] Lazari D. Freitas Leal J. Brock R. Bosman G. (2020). The relationship between aggregation and deformability of red blood cells in health and disease. *Front. Physiol.* 11:288. 10.3389/fphys.2020.00288 32351399 PMC7174766

[B15] Leonzino M. Reinisch K. M. De Camilli P. (2021). Insights into VPS13 properties and function reveal a new mechanism of eukaryotic lipid transport. *Biochim. Biophys. Acta* 1866:159003. 10.1016/j.bbalip.2021.159003 34216812 PMC8325632

[B16] Magnusson M. Eisfeldt J. Nilsson D. Rosenbaum A. Wirta V. Lindstrand A. (2020). Loqusdb: Added value of an observations database of local genomic variation. *BMC Bioinform.* 21:273. 10.1186/s12859-020-03609-z 32611382 PMC7329469

[B17] Murakami T. Abe D. Matsumoto H. Tokimura R. Abe M. Tiksnadi A. (2019). patient with McLeod syndrome showing involvement of the central sensorimotor tracts for the legs. *BMC Neurol.* 19:301. 10.1186/s12883-019-1526-9 31775676 PMC6882147

[B18] Park J. Halegoua S. Kishida S. Neiman A. M. (2015). A conserved function in phosphatidylinositol metabolism for mammalian Vps13 family proteins. *PLoS One* 10:e0124836. 10.1371/journal.pone.0124836 25915401 PMC4411106

[B19] Paucar M. Lindestad P. Å Walker R. H. Svenningsson P. (2015). Teaching video neuroimages: Feeding dystonia in chorea-acanthocytosis. *Neurology* 85 e143–e144. 26553947 10.1212/WNL.0000000000002108 PMC4653107

[B20] Peikert K. Dobson-Stone C. Rampoldi L. Miltenberger-Miltenyi G. Neiman A. De Camilli P. (2002). “VPS13A disease,” in *GeneReviews^®^ [Internet]*, eds Adam M. Feldman J. Mirzaa G. Pagon R. Wallace S. Bean L. (Seattle, WA: University of Washington). 20301561

[B21] Quick S. Heidrich F. Winkler M. Winkler A. Ibrahim K. Linke A. (2021). Cardiac manifestation is evident in chorea-acanthocytosis but different from McLeod syndrome. *Parkinsonism Relat. Disord.* 88 90–95. 10.1016/j.parkreldis.2021.05.015 34153885

[B22] Redman C. Huima T. Robbins E. Lee S. Marsh W. (1989). Effect of phosphatidylserine on the shape of McLeod red cell acanthocytes. *Blood* 74 1826–1835. 2790207

[B23] Reichel F. Kräter M. Peikert K. Glaß H. Rosendahl P. Herbig M. (2022). Changes in blood cell deformability in chorea-acanthocytosis and effects of treatment with dasatinib or lithium. *Front. Physiol.* 13:852946. 10.3389/fphys.2022.852946 35444561 PMC9013823

[B24] Sakuragi T. Nagata S. (2023). Regulation of phospholipid distribution in the lipid bilayer by flippases and scramblases. *Nat. Rev. Mol. Cell Biol.* 24 576–596. 10.1038/s41580-023-00604-z 37106071 PMC10134735

[B25] Shah P. Grewal U. Hamad H. (2023). *Acanthocytosis: StatPearls.* Treasure Island, FL: StatPearls Publishing. 31747195

[B26] Spesivtseva A. Gvarzhdec N. Autlev K. Kruchinin E. Kuznetsov I. (2023). Diseases of the neuroacanthocytosis group: A systematic review of clinical cases and difficulties in their diagnosis. *Adv. Life Sci.* 10 335–340.

[B27] Storch A. Kornhass M. Schwarz J. (2005). Testing for acanthocytosis A prospective reader-blinded study in movement disorder patients. *J. Neurol.* 252 84–90. 10.1007/s00415-005-0616-3 15654559

[B28] Stranneheim H. Lagerstedt-Robinson K. Magnusson M. Kvarnung M. Nilsson D. Lesko N. (2021). Integration of whole genome sequencing into a healthcare setting: High diagnostic rates across multiple clinical entities in 3219 rare disease patients. *Genome Med.* 13:40. 10.1186/s13073-021-00855-5 PMC7968334 33726816

[B29] Suzuki J. Imanishi E. Nagata S. (2014). Exposure of phosphatidylserine by Xk-related protein family members during apoptosis. *J. Biol. Chem.* 289 30257–30267. 25231987 10.1074/jbc.M114.583419 PMC4215210

[B30] Walker R. Danek A. (2021). “Neuroacanthocytosis” – overdue for a taxonomic update. *Tremor Other Hyperkinet. Mov.* 11:1. 10.5334/tohm.583 33510935 PMC7805383

[B31] Walker R. Peikert K. Jung H. Hermann A. Danek A. (2023). Neuroacanthocytosis syndromes: The clinical perspective. *Contact* 6:25152564231210339. 10.1177/25152564231210339 38090146 PMC10714877

