# Supplementary material for: Case report: Neuroacanthocytosis associated with novel variants in the VPS13A gene with concomitant nucleotide expansion for CANVAS and assessment with osmotic gradient ektacytometry
Source: Front Neurosci. 2024 Oct 2;18:1409366. doi: 10.3389/fnins.2024.1409366 (PMC11480079; doi:10.3389/fnins.2024.1409366)
Supplement: Supplementary file 1 [file Data_Sheet_1.pdf]

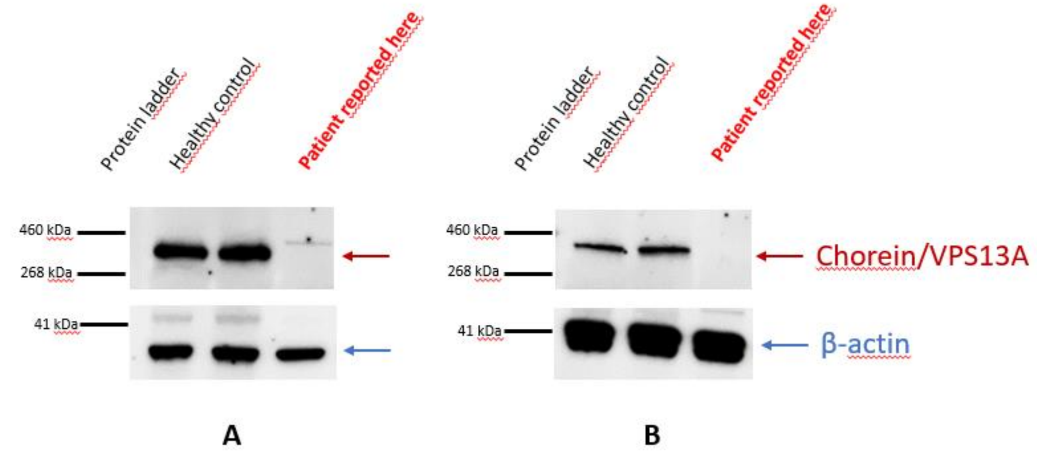

Figure e1

Chorein was absent in the sample from the patient reported here using two different antibodies, (A) antibodies from Sigma, (B) antibodies from Invitrogen.
